# A Case of Basal Cell Carcinoma Exacerbated with Akatsuki Disease

**DOI:** 10.3390/dermatopathology11040034

**Published:** 2024-11-22

**Authors:** Yuji Ohara, Issei Kido, Kozo Nakai

**Affiliations:** Department of Dermatology, Kochi Medical School, Kochi University, Kohasu, Oko-cho, Nankoku 783-8505, Kochi, Japan; jm-ohara@kochi-u.ac.jp (Y.O.); kido-issei@kochi-u.ac.jp (I.K.)

**Keywords:** Akatsuki disease, basal cell carcinoma

## Abstract

Akatsuki disease (also known as pomade crust) is characterized by skin lesions resulting from inadequate skin hygiene. It is sometimes influenced by underlying psychological factors. Akatsuki disease sometimes mimics cutaneous horn or skin cancer. However, there are no previous reports of skin cancer accompanied with Akatsuki disease. Herein, we report a 79-year-old woman who was referred to our department with a tumor on her left cheek. Before performing a biopsy, we recommended that her family assist with regular facial cleansing. Two months later, the scales and crusts on her entire face had disappeared and the tumor on the left cheek had reduced. Skin biopsy was performed, and histological examination revealed ulcerative basaloid lobules consisting of cells with a small cytoplasm and large hyperchromatic nuclei. Peripheral palisading and tumor-stroma clefting were observed. A diagnosis of basal cell carcinoma was made.

## 1. Introduction

Akatsuki disease was first reported by Sakamoto in 1964 in Japan. It was also known as pomade crust in the English literature in a report in 1975 [1]. Akatsuki disease is characterized by a skin surface with the accumulation of dirty exfoliation. It is defined as skin lesions induced by unpreferable skin hygiene condition, which may be influenced by underlying psychological factors. Herein, we report a case of basal cell carcinoma (BCC) that was clinically exacerbated with Akatsuki disease.

## 2. Case Report

**Figure 1:**
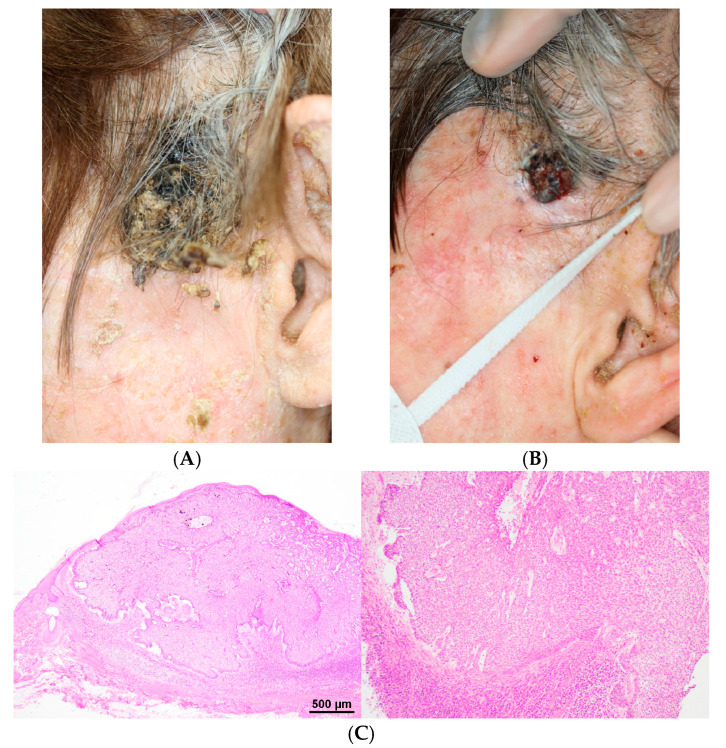
(**A**) Clinical presentation at first visit. A black mass of 80 mm in size is observed on the face. (**B**) Two months later, a black ulcerative nodule of 20 mm in size is observed. (**C**) Histological examination showing ulcerative basaloid lobules consisting of cells with a small cytoplasm and large, hyperchromatic nuclei (hematoxylin and eosin; left: ×40, right: ×100). Peripheral palisading and tumor-stroma clefting are observed.

A 79-year-old female was referred to our department with a tumor on the left cheek. Physical examination revealed a black mass of 80 mm in size (Figure 1A). We observed mild erythema accompanied by numerous scales and crusts covering her entire face. The patient had severe dementia and, as a result, had not washed her face for an extended period. Before biopsy, we advised her family to wash her face gently. The patient was treated with topical hydrophilic ointment and gentamicin. Two months later, the scales and crusts on her entire face had disappeared. Although the tumor on the left cheek was reduced, a black ulcerative nodule of 20 mm in size appeared (Figure 1B). Skin biopsy was performed, and histological examination revealed ulcerative basaloid lobules consisting of cells with a small cytoplasm and large, hyperchromatic nuclei. Peripheral palisading and tumor-stroma clefting were observed (Figure 1C). A diagnosis of BCC was made.

## 3. Discussion

It has been reported that Akatsuki disease mimics cutaneous horn or recurrent BCC [2,3]. To the best of our knowledge, there are no previous studies on skin cancer accompanied by Akatsuki disease. However, the presence of cutaneous horn-like pomade crusts may indicate underlying skin lesions such as actinic keratosis, warts, seborrheic keratosis, or skin cancer [4]. Therefore, biopsy and histological examination are necessary for accurate diagnosis. In general, histopathological examination of Akatsuki disease shows only ortho- and parakeratotic horn materials; therefore, to obtain the histological features of underlying skin lesions, biopsy should be performed after crust removal. In the present case, due to the patient’s severe dementia and her refusal of aggressive crust removal, it took two months to clear the crusts entirely through gentle washing.

In conclusion, this is the first report of BCC accompanied with Akatsuki disease. Biopsy is recommended after the removal of crusts to diagnose accurately the underlying skin lesions.

## Data Availability

No new data were created or analyzed in this study. Data sharing is not applicable to this article.
